# Apoptosis-Related Gene Expression in an Adult Cohort with Crimean-Congo Hemorrhagic Fever

**DOI:** 10.1371/journal.pone.0157247

**Published:** 2016-06-15

**Authors:** Nil Guler, Cafer Eroglu, Hava Yilmaz, Adil Karadag, Hasan Alacam, Mustafa Sunbul, Tom E. Fletcher, Hakan Leblebicioglu

**Affiliations:** 1 Department of Hematology, School of Medicine, Ondokuz Mayis University, Samsun, Turkey; 2 Department of Clinical Microbiology and Infectious Diseases, School of Medicine, Ondokuz Mayis University, Samsun, Turkey; 3 Department of Medical Microbiology, School of Medicine, Ondokuz Mayis University, Samsun, Turkey; 4 Department of Medical Biochemistry, School of Medicine, Ondokuz Mayis University, Samsun, Turkey; 5 Liverpool School of Tropical Medicine, Liverpool L3 5QA, United Kingdom; The University of Texas Medical Branch, UNITED STATES

## Abstract

Crimean-Congo Hemorrhagic Fever (CCHF) is a life threatening acute viral infection characterized by fever, bleeding, leukopenia and thrombocytopenia. It is a major emerging infectious diseases threat, but its pathogenesis remains poorly understood and few data exist for the role of apoptosis in acute infection. We aimed to assess apoptotic gene expression in leukocytes in a cross-sectional cohort study of adults with CCHF. Twenty participants with CCHF and 10 healthy controls were recruited at a tertiary CCHF unit in Turkey; at admission baseline blood tests were collected and total RNA was isolated. The RealTime ready Human Apoptosis Panel was used for real-time PCR, detecting differences in gene expression. Participants had CCHF severity grading scores (SGS) with low risk score (10 out of 20) and intermediate or high risk scores (10 out of 20) for mortality. Five of 20 participants had a fatal outcome. Gene expression analysis showed modulation of pro-apoptotic and anti-apoptotic genes that facilitate apoptosis in the CCHF patient group. Dominant extrinsic pathway activation, mostly related with TNF family members was observed. Severe and fatal cases suggest additional intrinsic pathway activation. The clinical significance of relative gene expression is not clear, and larger longitudinal studies with simultaneous measurement of host and viral factors are recommended.

## Introduction

Crimean Congo Hemorrhagic Fever (CCHF) is a life threatening acute viral infection caused by a RNA virus belonging to the family *Bunyaviridae*. The first documented infection was observed in 1944 in the Crimea region of Ukraine and was designated as Crimean haemorrhagic fever. It was later observed in Congo in 1956 and the name was changed to CCHF [1]. CCHF virus (CCHFV) is an enveloped single-stranded RNA virus, the genome of which includes small (S), medium (M) and large (L) segments, and phylogenetic analysis of CCHFV strains has revealed high degree of genetic diversity amongst strains, particularly between viruses from different geographic regions [2].

CCHF is a major emerging infectious disease threat and is widely distributed across Africa, Eastern Europe, Asia and the Middle East. Turkey reports up to 1000 cases per year and CCHFV is predominantly transmitted to humans via *Hyalomma* spp. ticks [3]. Clinical features of disease represent a spectrum of disease severity, characterized by fever, bleeding and lethargy, and has a case fatality rate of 5–50%. The most common hematologic findings in CCHF are leukopenia (60%) and thrombocytopenia (100%)[4], with evidence of haemophagocytosis in the bone marrow [5]. At present there is no approved vaccine or therapeutic agent, although ribavirin and hyper immune serum are utilized by some clinicians.

The pathogenesis of CCHF remains poorly understood but apoptotic programmed cell death has been proposed to play a role [2]. Regulation of apoptosis through suppression or induction during acute viral infection is important for virus survival and dissemination [6]. Apoptosis can be started by an extrinsic (death receptor mediated) or intrinsic pathway (mitochondria mediated). The external pathway is activated when ligands—TNF-α, TNF-related apoptosis-inducing ligand (TRAIL), and Fas ligand (FasL) known as CD95L, bind to death receptors on the cell surface. Some TRAIL receptors do not contain death domains and are referred to as decoy receptors 1 (DcR1) (TNFRSF10C) and 2 (DcR2) (TNFRSF10D) and prevent apoptosis signals from initiating. The intrinsic pathway to cell death is controlled by interactions on the mitochondrial outer membrane between sub-groups of the BCL-2 family, including the effector proteins BAX and BAK [7]. Both pathways ultimately result in up-regulation of effector caspases (caspase-3 & caspase-7) resulting in fragmentation of the DNA, cytoskeleton, nuclear proteins and expression of ligands for phagocytic cells [8].

Apoptosis has been demonstrated in a range of cells in other acute viral infections including Dengue [9]. Massive intravascular apoptosis, including leukocyte apoptosis, has also been demonstrated in Ebola virus infection [10], where it has been shown to be associated with a fatal outcome. CCHFV has been shown to induce caspase-3 and modulate both intrinsic and extrinsic pathways in cell cultures [11,12]. Elevated apoptotic markers have also recently been demonstrated in a paediatric cohort of CCHF [13], and associations were found with the disease’s severity criteria. We have previously observed apoptotic transformation in leukocytes in peripheral blood smears of patients with CCHF, and in the present work, this was investigated further by studying up and down-regulation of genes which are involved in leukocyte apoptosis in a cohort of adult patients with CCHF.

## Methods

### Study Participants

Participants in the study were recruited from a designated CCHF tertiary referral hospital in Turkey between May and August 2011. Hospitalized patients with suspected CCHF and healthy controls were recruited into the study with CCHF confirmed through viral nucleic acid detection by polymerase chain reaction (PCR) of plasma in the Ministry of Health regional reference laboratory in Samsun. Patients with a negative PCR result were excluded from further analysis. All participants were free from antiviral drugs and immunotherapy for CCHF during blood sampling. Demographic, epidemiological and clinical data were systematically extracted from the medical records of all participants with confirmed CCHF. Participants had a CCHF severity grading score (SGS) performed in accordance with Bakir et al [14] at admission, and were classified into three risk groups for mortality: low (0–4 points), intermediate (5–8 points), and high (9–14 points) (S1 and S2 Tables). The CCHF SGS was established using several clinical and laboratory variables that were assumed to be associated with mortality and had clinical importance. It has subsequently been validated and may be used to aid triage/risk stratification of patients and to improve the functionality of healthcare staff.

### Patient sampling and RealTime ready Apoptosis Panel

Four milliliter blood samples were drawn into EDTA blood tubes on the first day of hospitalization and standard baseline blood tests took place (Table 1). Red Blood Cell Lysis Buffer (Roche Diagnostics, Germany) was added to the blood samples and total RNA was isolated utilizing a High Pure RNA Isolation Kit (Roche Diagnostics, Germany). RNA samples were then stored at kept at -70°C until sample processing when cDNAs was obtained using a Transcriptor First Strand cDNA Synthesis Kit (Roche Diagnostics, Germany). Expression levels of apoptotic-related genes were determined using The RealTime ready Human Apoptosis Panel (Roche Diagnostics, Germany) and a LightCycler 480 Probes Master. The RealTime ready Apoptosis Panel consists of ready-to-use qPCR assays designed for expression profiling of genes involved in the apoptosis pathway. Each multi-well plate contains assays for 84 different apoptosis-related genes and seven housekeeping genes, as well as five controls. This study was approved by the Ondokuz Mayis University Ethical Committee (2010/178) and all patients provided written informed consent.

**Table 1:** Participant laboratory characteristics according to severity score: Mean (Minimum-Maximum) ±Standard Deviation.

Variable (reference range) units	Low risk (n = 10)	Intermediate risk (n = 8)	High risk (n = 2)
Age years	50.4 (39–63)±8	56.8 (20–73)±21	67 (63–71)±6
Blood Urea Nitrogen (5–24) mg/dL	14.3 (8.6–24)±5	21 (6.5–44)±12	40.5 (30–51.4)±15
Creatinine (0.4–1.4) mg/dL	0.85 (0.55–1.04) ±0.2	1.1 (0.53–1.9) ±0.4	1.82 (1.81–1.84) ±0.02
Lactic dehydrogenase (LDH) (140–280) IU/L	370 (183–567) ±132	1731 (705–5067) ±1446	3122 (2447–3797) ±955
Aspartate transaminase (AST) (5–40) IU/L	119 (16.4–279) ±83	722 (128–2198) ±674	1299 (1087–1511) ±300
Alanine transaminase (ALT) (7–56) IU/L	66 (16–209) ±57	237 (24–547) ±177	501 (406–596) ±134
Hemoglobin(12–15) gr/dL	14 (11.3–15.6) ±1.3	14.5 (12.2–18.3) ±2	11.6 (9–14.2) ±4
Platelet count (150–450) x10^9^ cells/L	103 (52–212) ±54	25.6(12–52) ±16	21 (10–32) ±16
PT(11–14) seconds	11.9 (10.8–12.9) ±0.8	14 (10.2–17.4) ±2	22.65 (13.3–32) ±13
PTT (25–35) seconds	35.5 (27–43) ±6	55 (31.3–72.4) ±14	84.5 (77–92) ±11
International normalized ratio (INR)	1.05 (0.96–1.16) ±0.10	1.3 (0.91–1.57) ±0.20	2.08 (1.19–2.96) ±1.0
Leucocyte (3.9–11.7) x10^9^/L	2.39 (0.94–5.00) ±1.18	5.73 (1.89–9.89) ±3.19	10.91(7.74–14.08) ±4.48
Neutrophil (1.9–7.9) x10^9^/L	1.61(0.59–3.00) ±0.76	4.51 (1.53–8.50) ±2.68	7.92 (6.24–9.60) ±2.38
Lymphocyte(1.3–3.6) x10^9^/L	0.571 (0.27–1.59) ±0.38	0.80 (0.30–1.83) ±0.58	2.26(0.88–3.63) ±1.95
Monocyte (0.2–0.5)x10^9^/L	0.11 (0.20–0.24) ±0.08	0.135(0.07–0.22) ±0.06	0.28 (0.15–0.41) ±0.18
Basophil(0–0.1) x10^9^/L	0.02(0.01–0.07) ±0.02	0.01(0.02–0.03) ±0.01	0.062 (0.02–0.10) ±0.06
Eosinophil (0–0.4)x10^9^/L	0.03 (0.01–0.06) ±0.02	0.012(0.01–0.04) ±0.01	0.02(0.02–0.02) ±0.02

### Statistical analyses

The REST 2009 program (Qiagen, Germany) was used to quantify changes in the expression levels of genes and to determine statistical differences between the CCHF and control groups, and between CCHF fatal and survivor groups. Differences in gene expression were also analyzed between intermediate or high SGS risk score participants (Group 1) and low SGS risk score (Group 2). P values less than or equal to 0.05 were accepted as statistically significant. Descriptive analysis of patient characteristics was performed using the Statistical Package for the Social Sciences (SPSS) version 15 for Windows (SPSS Inc., Chicago, IL, USA).

## Results

Twenty patients (female: 6; male: 14) and 10 healthy controls (female: 5; male: 5) were included in this study. The patients mean age was 54.6 (20–73) years-old. At admission, participants were a mean 3.4 (1–6) days since onset of symptoms of disease. According to the SGS system 10 participants were low risk (score 0–4), 8 participants intermediate risk (5–8), and 2 participants in the high risk group (9–14). Five of the 20 participants (25%) died of CCHF during the hospital admission. Three of the 5 fatal cases had an SGS score of 8, 1/5 a score of 9 and 1/5 a score of 13. Petechiae and/or ecchymosis were detected in 7/20 (35%) participants, and significant haemorrhage developed in a further 6/20 (30%) participants.

Detailed laboratory characteristics are displayed in Table 1 stratified by severity group. Mean cohort data (n = 20) at baseline showed: leukocytes 4.58 x 10^9^ cells/L (0.94–14.1); neutrophils 3.4 x 10^9^ cells/L (0.59–9.6); lymphocytes 0.83 x 10^9^ cells/L (0.03–3.63); monocytes 0.14 x10^9^ cells/L (0.02–0.41); basophils 0.11 x10^9^ cells/L (0.01–1.01); alanine aminotransferase (ALT) 178 IU/L (16–596); aspartate transaminase (AST) IU/L 410 (16.4–2198); lactate dehydrogenase (LDH) 1233 IU/L (183–5067); platelets 69.7 x 10^9^/L (10–212); prothrombin time (PT) 13.8 seconds (10.2–32); partial thromboplastin time (PTT) 48.2 seconds (26.6–92); international normalized ratio (INR) 1.24 (0.91–2.96); blood urea nitrogen (BUN) 19.6 mg/dL (6.5–51.4); and creatinine 1.05 mg/dL (0.53–1.9). The healthy control group exhibited a normal profile in laboratory parameters.

Expression analysis showed that the BCL2L1, BCL2L2, BIRC5, CASP2, CASP3, SOCS2, TNFSF10, and TRAF3 genes were up-regulated and the BCL2L10, BID, CFLAR, MCL1, NFKB2, PTEN, STAT5B, TNFRSF10C, TNFRSF10D, TNFRSF1B, and TRAF1 genes were down-regulated in the patient group compared with the healthy control group (Table 2).

**Table 2:** Changes in gene expression level (up- or down-regulation) in the patient group compared with the healthy group.

Gene	Reaction Efficiency	ExpressionTimes	Std. Error	Results	95% C.I.	P-value
BCL2L1	1,0	2,125	0,850–6,082	UP	0,262–11,940	0,011
BCL2L10	1,0	0,128	0,012–3,619	DOWN	0,002–17,597	0,015
BCL2L2	1,0	3,373	1,947–9,525	UP	0,007–24,859	0,028
BID	1,0	0,251	0,117–0,866	DOWN	0,001–1,753	0,005
BIRC5	1,0	17,412	2,827–70,762	UP	1,224–110,335	0,000
CASP2	1,0	1,474	0,765–2,667	UP	0,404–6,175	0,047
CASP3	1,0	2,903	0,952–11,071	UP	0,028–32,494	0,028
CFLAR	1,0	0,468	0,207–1,012	DOWN	0,068–2,369	0,003
MCL1	1,0	0,336	0,127–0,817	DOWN	0,014–1,622	0,005
NFKB2	1,0	0,289	0,146–0,628	DOWN	0,037–0,990	0,000
PTEN	1,0	0,227	0,120–0,707	DOWN	0,000–1,492	0,009
SOCS2	1,0	6,069	1,772–19,478	UP	0,688–86,897	0,000
STAT5B	1,0	0,188	0,029–0,674	DOWN	0,002–1,168	0,006
TNFRSF10 C	1,0	0,047	0,012–0,262	DOWN	0,000–0,822	0,001
TNFRSF10 D	1,0	0,452	0,151–1,588	DOWN	0,063–4,274	0,035
TNFRSF1B	1,0	0,378	0,193–0,847	DOWN	0,065–1,706	0,001
TNFSF10	1,0	1,992	0,768–4,350	UP	0,247–22,329	0,039
TRAF1	1,0	0,440	0,128–1,319	DOWN	0,067–3,004	0,023
TRAF3	1,0	1,978	0,853–4,860	UP	0,287–20,458	0,027

There were no significant changes in the expression of AKT1, APAF1, AVEN, BAD, BAG1, BAK1, BAX, BBC3, BCL2, BCL2L11, BCL2L13, BIK, BIRC2, BIRC3, BOK, CAD, CASP1, CASP10, CASP4, CASP5, CASP6, CASP7, CASP8, CASP8AP2, CASP9, CRADD, DFFA, DIABLO, endoG, FADD, FAM96A, FAM96B, FAS, FASLG, HMGB1, HRK, HSP90B1, HTRA2, LRDD, NFKB1, NGFR, PMAIP1, REL, RELA, RELB, SOCS3, STAT1, STAT5A, TNF, TNFRSF10 A, TNFRSF10 B, TNRFSF1A, TNFRSF25, TNFSF8, TP53, TP53I3, TRAF2, TRAF5, TRAF6, and TRAF7 genes in the patient group compared with the healthy control group.

Changes in the expression of apoptosis-related genes between fatal and survivor group are shown in Table 3. Three genes showed significant down-regulation in expression in fatal cases compared to survivors (BBC3, BCL2L2 and CASP2), and one gene significant up-regulation (TNFRSF1A).

**Table 3:** Changes in gene expression level (up- or down-regulation) in fatal cases compared to survivors.

Gene	Reaction Efficiency	Expression times	Std. Error	Results	95% C.I.	P-value
BBC3	1,0	0,423	0,137–1,093	DOWN	0,057–1,896	0,015
BCL2L2	1,0	0,121	0,002–0,862	DOWN	0,001–1,557	0,002
CASP2	1,0	0,546	0,325–0,916	DOWN	0,177–1,704	0,006
TNFRSF1A	1,0	3,681	0,909–17,450	UP	0,165–35,533	0,036

Changes in the expression of apoptosis-related genes between the intermediate/high SGS risk group and low SGS risk group participants are shown in Table 4. Expression analysis showed that the BAX, BCL2L13, CASP4, CASP9, CRADD, ReIA, TNFRSF10C and TNFRSF1A genes were up-regulated and the BCL2L10 and BCL2L11 genes were down-regulated.

**Table 4:** Changes in gene expression level (up- or down-regulation) in intermediate and high risk participants compared with low risk participants.

Gene	Reaction Efficiency	Expression times	Std. Error	Results	95% C.I.	P-value
BAX	1,0	2,110	0,824–5,344	UP	0,340–14,112	0,027
BCL2L10	1,0	0,101	0,004–2,043	DOWN	0,001–26,704	0,030
BCL2L11	1,0	0,049	0,002–1,603	DOWN	0,000–28,436	0,013
BCL2L13	1,0	2,876	0,685–12,136	UP	0,215–44,091	0,033
CASP4	1,0	8,341	0,845–104,284	UP	0,036–611,645	0,024
CASP9	1,0	1,707	0,907–3,446	UP	0,465–6,325	0,030
CRADD	1,0	7,342	0,676–252,737	UP	0,273–1005,402	0,026
RelA	1,0	5,626	0,775–59,557	UP	0,085–130,134	0,018
TNFRSF10C	1,0	5,898	0,764–36,234	UP	0,150–2406,870	0,020
TNFRSF1A	1,0	3,984	1,077–17,458	UP	0,227–32,879	0,008

## Discussion

The pathogenesis of CCHF remains poorly understood despite significant recent scientific progress and research efforts [2]. Viral factors and an impaired host immune response including an exaggerated pro-inflammatory cytokine effect, all play a role in the severity and prognosis of viral haemorrhagic fevers including CCHF [15,16]. Direct cytopathic effects of the virus contribute to disease severity, but the targeting of the innate immune response, combined with a subsequent failure in adaptive immunity, exacerbated by lymphocyte apoptosis, play key roles in pathogenesis and outcome [17]. In this study, we aimed to evaluate the role of apoptosis in CCHF, through expression and regulation of apoptotic genes in an adult cohort in Turkey. The cohort had an ideal balance of severe/fatal and moderate/mild CCHF cases to investigate apoptotic change and a large panel of apoptotic genes from different pathways were evaluated.

The gene of caspase-3, that is an important executioner caspase in apoptosis, was up-regulated in our study compared to healthy volunteers. With respect to the extrinsic pathway, one of its most important ligands TRAIL (TNF-related apoptosis inducing ligand), that induces apoptosis, was up-regulated. Decoy receptors of TRAIL, DcR1 and DcR2 were down-regulated resulting in cells being more sensitive to apoptosis [18]. Another important finding related to cell sensitivity to apoptosis was the down-regulation of c-FLIP expression. The c-FLIP protein structurally resembles caspase-8 and effects the extrinsic pathway in opposite ways dependent on the extent of its expression. Down-regulated and low levels of c-FLIP are thought to promote caspase-8 activation, whereas in high concentrations c-FLIP may compete for binding, reduce caspase-8 activity and the cell’s susceptibility to apoptosis [7,19].

Analysis of the intrinsic apoptotic pathway related genes, showed up-regulation of anti-apoptotic BIRC5 [20] (an inhibitor of apoptosis protein) and BID, a pro-apoptotic member of the Bcl-2 family was down-regulated compared to controls. Protein kinase B (AKT) plays a key role in cell survival including inhibition of apoptosis and cell proliferation. Phosphatase and tensin homolog (PTEN) which is a critical regulator of AKT [21] was also found to be down-regulated. No significant differences in gene expression were noted in BAX or DIABLO genes, which are important components of the intrinsic pathway. These findings combined with BIRC5, PTEN and BID results do not support major intrinsic apoptotic pathway activation in CCHF patients compared to controls and the up-regulation demonstrated in caspase-2 may be independent [22].

NF-κB is a major rapidly acting transcription factor that regulates genes responsible for both the innate and adaptive immune response, including apoptotic genes affecting both the intrinsic and extrinsic pathways [23]. NFκB exerts its anti-apoptotic effect via two pathways, the canonical and non-canonical that both result in mature dimeric NFκB proteins that translocate to the cell nucleus and activate anti-apoptotic genes. The NFκB system is composed of five subunits, which are RelA, RelB, c-Rel, p50 monomer (encoded by the NFκB1 gene) and p52 monomer (encoded by the NFκB2 gene) [24]. While the p50: RelA, NFκB dimer is dominant in canonical pathway, the p52: RelB, NFκB dimer is dominant in non-canonical pathway.

There was significant down-regulation in NFκB2 gene expression whilst there were no changes observed for RelA, RelB, c-Rel, and p50. We interpreted this result as inhibition of the non-canonical NFκB pathway because of down regulation of its dominant member NFκB2. These finding are also consistent with our results as the canonical pathway is also thought to be predominantly involved in the regulatory process of the intrinsic apoptotic pathway [23]. Interactions also exist between NFκB and c-FLIP and the transcription of c-FLIP is positively regulated by NFκB. NFκB levels are also increased by the interaction of c-FLIP with the TRAF-1 (TNF receptor-associated factor-1) and TRAF-2 [25,26]. TRAF-1 expression was found to be down-regulated in our study and TRAF 3, that has a critical negative regulatory role in non-canonical NFκB signaling [24,27], was up-regulated.

The JAK (Janus tyrosine kinase)-STAT (Signal Transducer and Activator of Transcription) pathway is an important cell signaling cascade that is activated by cytokines and their receptors. Activation of STAT initiates synthesis of SOCS (suppressors of cytokine signaling) negatively regulates STAT signaling by serving as a negative feedback loop [28]. Compared to healthy controls STAT5B gene expression was down-regulated in CCHF combined with up-regulation of SOCS2, indicating a direct cytokine effect. As previously discussed, the up-regulated TRAIL and TRAF-3 and down-regulated TRAF-1, DcR1 and DcR2, all belong to the TNF family and are implicated in apoptosis in CCHF. Its receptor, TNFRSF1B that also serves as an inhibitor of TNF-related apoptosis [29,30], was also found to be down-regulated in our study. The role of pro-inflammatory cytokines and a ‘cytokine storm’ in CCHF disease severity are increasingly recognized, with TNF-α expression linked to clinical severity and mortality [16]. Our results support this hypothesis suggesting additional effects through cytokine mediated apoptosis.

Previous research has implicated important clinical and laboratory factors that are related to mortality in CCHF [31]. As a result, as well comparing gene expression in CCHF and healthy volunteers acting as controls, we wanted to evaluate the differences further through sub-group analysis of fatal and non-fatal CCHF cases and by stratifying for CCHF disease severity. There were fewer significant differences but TNFRSF1A (TNF receptor superfamily member 1A) was up-regulated in the fatal CCHF group. This receptor has a key role in TNF-related apoptosis [21,22]. PUMA (p53 up-regulated modulator of apoptosis), also known as BBC3 (BCL-2 binding component), was down-regulated and the down-regulation of caspase-2 gene expression in the fatal group may be related to p53 [32]. BCL-2 is a regulatory protein that may induce or inhibit apoptosis and Baize et al. elicited interesting results when evaluating BCL-2 levels in Ebola virus infection. Increased synthesis of BCL-2 mRNA was detected at the time of T-cell activation in survivors in their study, with disappearance of BCL-2 mRNA expression observed in fatal cases [10].

In our study, there was no difference between BCL-2 gene expression between fatal and non-fatal cases. However, BCL2L2 which is another anti-apoptotic protein was down-regulated in the fatal CCHF group compared to survivor group. In contrast it was up-regulated in the larger cohort when compared to the healthy volunteers. BLC2L2 (also known as BCL-W) is one of the pro-survival proteins of the BCL-2 family and these contrasting results, in combination with the down-regulation of PUMA, suggest modulation of the intrinsic pathway in fatal cases, and are interesting targets for further research. Intrinsic pathway activation linked to CCHF disease severity was also suggested by gene expression analysis of the intermediate/high SGS risk group compared to the low SGS risk group. There was up-regulation of pro-apoptotic BAX, BCL2L13, CASP 9 and down-regulation of anti-apoptotic BCL2L10 (that suppresses apoptosis) in the intermediate and high risk groups. CRADD which acts with caspase-2 in intrinsic pathway activation [33] was found up-regulated. In the intermediate/high risk CCHF group there was also evidence of extrinsic pathway activation, with upregulated caspase-4, that has a role in apoptosis induced by TRAIL [34], and up-regulation of the pro-apoptotic TNFRSF1A gene, and decoy receptor TNFRSF10C gene.

To our knowledge, this is the first *in vivo* study to describe the apoptotic gene expression in leukocyte in CCHF and supports our previous observations in CCHF patients. Leukocyte apoptosis may be responsible for the cytopenia observed in CCHF, and apoptotic signals will also most likely affect myeloid cells in bone marrow. During apoptosis, cells express ligands for phagocytic cells and trigger phagocytosis, and the observed haemophagocytosis in bone marrow may be a further clue to apoptosis in the bone marrow in CCHF. Apoptotic processes may also be more widespread targeting liver, brain and endothelial cells [35], and the multi-organ failure seen in CCHF may be as a result of haemophagocytic activity triggered by apoptosis in several organs. One of the limitations of the study is that we did not evaluate apoptotic gene expression individually by leukocyte subtypes. Given the heterogeneous nature of the clinical disease a larger sample size, particularly in the fatal and survivor group would have increased the studies power to detect differences. In addition, we utilized the SGS tool for grading severity of CCHF and in subsequent analysis by group. Although this has been validated as a useful prognostic tool it is clear that SGS has its own limitations, relates to a specific disease time-point and could be improved and refined incorporating variables such as CCHFv viral load. We also only report cross-sectional gene expression data at one time-point during an early stage of CCHF clinical disease. A longitudinal study design with additional sampling points would have provided important additional data, and in the future novel methods such as high resolution sequencing will provide further insights. This must be balanced against the practical difficulties and laboratory safety requirements of undertaking research in patients with viral haemorrhagic fever.

It must also be recognized that although the values we have reported may be statistically significant, the clinical significance of the relative expression of particular genes is not clear. A 2-fold increase in expression may be a very important change for some genes and relatively insignificant for others, regardless of statistical significance. Although we have investigated our results linked to patient outcomes and CCHF severity grading score (SGS), these are broad measures highlighted by the fatal cases that occurred in the intermediate group. It must also be appreciated that as per the clinical disease in CCHF there is probably a spectrum of apoptosis in CCHF dependent on disease stage and disease severity. Improved understanding of apoptotic processes in CCHF could be achieved through simultaneous measurement of host and viral factors including cytokine response, quantitative viral load and in depth genomic sequencing in cohorts with a standardized platform of supportive care.

Further research is also required to determine the broader role of apoptosis in the pathogenesis of acute viral infection and CCHF. In particular research to improve the understanding of the specific viral and host immune response factors that initiate apoptotic signaling are key, and must be correlated with clinical data. Until this relationship is more fully understood it is not clear if novel translational approaches targeting the pathways of apoptosis would improve immune mediated viral clearance or aid in viral replication and dissemination. In conclusion, our study of leucocytes in patients with CCHF provides further evidence of apoptosis in the disease and suggests dominant extrinsic pathway activation, mostly related with TNF family members, but additional intrinsic pathway activation in severe/fatal cases.

## Supporting Information

S1 Table Participant SGS groups. (XLS)

S2 Table Participant epidemiological and baseline clinical data. (XLS)
